# LncRNA LINC01833 is a Prognostic Biomarker and Correlates with Immune Infiltrates in Patients with Lung Adenocarcinoma by Integrated Bioinformatics Analysis

**DOI:** 10.1155/2023/3965198

**Published:** 2023-01-27

**Authors:** Wei Liu, Qiu Wan, Enzhu Zhou, Ping He, Lixin Tang

**Affiliations:** ^1^Department of Emergency Eedicine, Chongqing Public Health Medical Center, Shapingba 400000, Chongqing, China; ^2^Department of Respiratory Geriatrics, Chongqing Public Health Medical Center, Shapingba 400000, Chongqing, China; ^3^Department of Thoracic Surgery, Chongqing Southwest Hospital, Shapingba 400000, Chongqing, China

## Abstract

Due to the absence of accurate tools for early detection and successful treatment, lung adenocarcinoma (LUAD) is one of the most aggressive tumors with high morbidity and mortality globally. It is absolutely necessary to investigate the process behind its development and search for new biomarkers that could aid in the early detection of LUAD. There is a correlation between the immune microenvironment of the tumor and the prognosis of lung cancer as well as the efficacy of immunotherapy. Long noncoding RNAs (lncRNAs) have been identified as potential prognostic biomarkers linked to immunological activities. In this study, we identified 1 downregulated lncRNA and 76 upregulated lncRNAs in LUAD samples from TCGA datasets. Among the 77 dysregulated lncRNAs, our attention focused on lncRNA LINC01833 (LINC01833). When compared with nontumor specimens, the level of expression of LINC01833 was shown to be significantly elevated in LUAD samples. In addition, the data of the ROC study revealed that LUAD patients with high LINC01833 expression had an AUC value of 0.840 (95% confidence interval: 0.804 to 0.876). There was a correlation between high LINC01833 expression and an advanced clinical stage. Patients who had a high expression of LINC01833 were shown to have a lower overall survival rate (*p* < 0.001) and a lower disease-specific survival rate (*p* = 0.004) in comparison to patients who were in the low LINC01833 group, according to the data on survival. In addition, the results of the multivariate analysis revealed that high LINC01833 expression was an independent predictor of poor survival in LUAD. Moreover, the immune analysis revealed that we found that the expression of LINC01833 was positively associated with Th2 cells, aDC, and Tgd, while negatively associated with Mast cells, Tcm, Eosinophils, iDC, DC, Tem, Th17 cells, and pDC. Overall, our data point to the possibility that the unique lncRNA LINC01833 might be employed as a diagnostic and prognostic marker, and as a result, it has a significant impact on clinical practice.

## 1. Introduction

Lung cancer is a leading cause of cancer-related death worldwide [1]. Non-small-cell lung cancers account for around 85% of all occurrences of lung cancer, with lung adenocarcinoma (LUAD) being the most frequent histologic subtype of non-small-cell lung cancer and representing approximately 40% of all lung cancer cases [2, 3]. Even though significant strides have been made in the detection and treatment of lung cancer, such as the development of molecularly targeted therapy medicines and the application of low-dose computed tomography, the 5-year survival rate for patients with lung cancer is only 17.4% [4, 5]. Because we have such a poor grasp of the pathophysiology of LUAD, there are very few reliable prognostic signs. Although medicines targeting EGFR, TP53, AKT1, KRAS, and PTEN have been widely deployed in patients with advanced lung cancer, their clinical promise is still restricted [6, 7]. This is because these genes typically undergo mutations and copy number variations in LUAD. A good approach for forecasting patient survival status is required in order to facilitate the diagnosis of early-stage LUAD and the treatment of various patients with realistic treatment regimens [8, 9]. It is necessary to avoid wasting medical resources and prolonging the conditions of patients.

Epigenetics is a biological study method that investigates changes in phenotype that are not based on DNA sequence. These changes include DNA methylation, histone modification, chromatin remodeling, and noncoding RNA [10, 11]. Epigenetics is also known as chemical genetics. Long noncoding RNAs (lncRNAs) are a type of noncoding RNA with >200 nucleotides in length [12]. In a wide variety of biological pathways and cellular processes, such as chromosome inactivation and differentiation, the reprogramming of stem cell pluripotency, and the modulation of invasion and apoptosis, it is thought that lncRNAs play crucial regulatory roles [13, 14]. One example of this is the reprogramming of stem cell pluripotency. Recent findings have demonstrated that thousands of aberrant lncRNAs are strongly associated with a range of cancer types, including LUAD [15, 16]. For example, earlier research has shown that many lncRNAs are dysregulated and play crucial roles in the course of LUAD; these lncRNAs include lncRNA LINC00525, lncRNA UPLA1, lncRNA ZFPM2-AS1, lncRNA HMMR-AS1, lncRNA TGFB2-AS1, etc. [17–21]. However, many LUAD-related lncRNAs have not been identified.

LncRNA LINC01833 (LINC01833) was recently discovered (lncRNA). It was only infrequently documented that it had a function and potential in malignancies. In the past, a number of studies have revealed that the expression of LINC01833 was misregulated in several malignancies, including endometrial cancer, bladder cancer, and lung cancer [22–24]. However, its clinical significance in LUAD has not been investigated.

## 2. Materials and Methods

### 2.1. Data Collection

Using the TCGA database (https://tcga-data.nci.nih.gov/tcga/), we were able to gather clinical data as well as FPKM RNA-seq data from LUAD cases. These cases included 535 cancer samples and 59 noncancerous samples. The next thing we did was match the clinical data of patients with their transcriptome data, using the patient ID as the key. Patients who had medical records or IDs that did not match were not included in this study.

### 2.2. Identification of Differentially Expressed lncRNAs

We compared LUAD specimens and nontumor specimens using the limma program in Bioconductor, which is part of the R software. Based on this comparison, we selected the differentially expressed lncRNAs (DElncRNAs). The thresholds that were used to identify DElncRNAs were |log2 (fold change, FC)| greater than 5, and the false discovery rate (FDR) was less than 0.05.

### 2.3. Tumor Immune Infiltration Analysis

The XIANTAO platform (https://www.xiantao.love/) was used to conduct an analysis of the immune infiltration profiles of the tumor. In order to differentiate between the various immunocytes, a total of 24 immunological markers were utilized. Using the single-sample generalized estimating equations analysis (ssGSEA) method, we were able to determine the Spearman correlations between immunocyte markers and lncRNA expression levels [25].

### 2.4. Statistical Analysis

The comparison of the expressions of LINC01833 between LUAD samples and nontumor samples was performed using the two-sample Student's *t*-test. The relationships between clinical parameters and the expression of LINC01833 were calculated using *χ*^2^ tests. Kaplan–Meier analysis was utilized to demonstrate the prognostic value of LINC01833 expression in the TCGA cohort, and the log-rank test was utilized to evaluate the statistical significance of the findings. Analyses using both univariate and multivariate methods were carried out with Cox's proportional hazard regression model in order to determine whether or not LINC01833 expression was dependent on other clinical factors. In order to characterize the predictive accuracy of LINC01833, the time-dependent ROC curve and the AUC were utilized. The entire statistics were completed using the R program 3.6.2. A *p* value of <0.05 was considered to indicate a statistically significant difference.

## 3. Results

### 3.1. The Expression of LINC01833 was Downregulated in LUAD Patients

**Figure 1:**
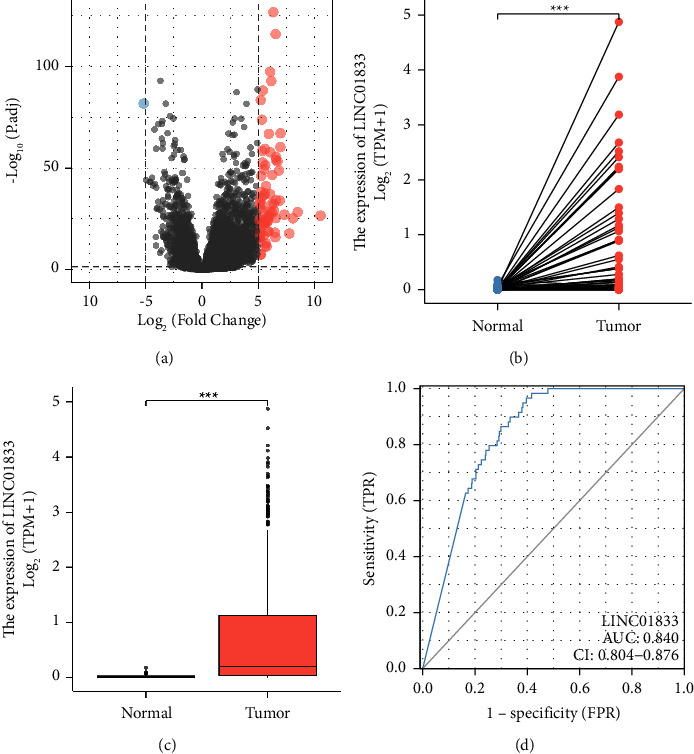
The expression of LINC01833 in LUAD patients. (a) A heat map of the differentially expressed lncRNAs between LUAD specimens and nontumor specimens. (b and c) The expressions of LINC01833 were increased in LUAD specimens based on TCGA datasets. (d) ROC curve for LINC01833 as a diagnostic marker for LUAD specimens. ^∗∗∗^*p* < 0.001.

To screen the dysregulated lncRNAs in LUAD, we analyzed TCGA datasets. As shown in Figure 1(a), we identified one downregulated lncRNA and 76 upregulated lncRNAs. Among the differentially expressed lncRNAs, our attention focused on LINC01833, which was highly expressed in LUAD specimens compared with nontumor specimens (Figures 1(b) and 1(c)). In addition, this lncRNA is rarely reported in tumors. The diagnostic utility of LINC01833 for patients with LUAD was subsequently investigated. According to the ROC assays, high LINC01833 expression yielded an AUC value of 0.840 for OS, with a 95% confidence interval ranging from 0.804 to 0.876 (Figure 1(d)). Based on our findings, LINC01833 had the potential to serve as an indicator for the diagnosis of patients with LUAD.

### 3.2. Correlation of LINC01833 Expression in LUAD with Clinicopathological Features

**Figure 2:**
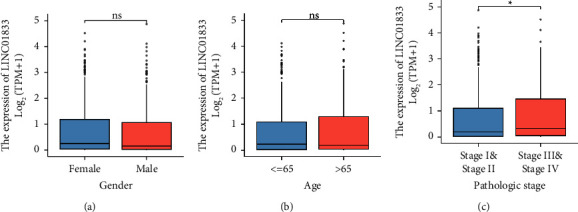
The association between LINC01833 and several clinical factors, including (a) gender, (b) age, and (c) clinical stage.

Next, we investigated whether or not there was a connection between the expression of LINC01833 and the numerous clinicopathological variables that were presented in LUAD patients. As can be seen in Figures 2(a) and 2(b), we did not discover any significant differences in the expression of LINC01833 between patients who were female, patients who were male, patients who were under the age of 65, and patients who were over the age of 65. However, our group observed that the expression of LINC01833 was increased in LUAD samples with advanced stages compared with LUAD samples with early stages (Figure 2(c)). Patients whose LINC01833 expression was greater than the average were placed in one group, referred to as the high expression group; patients whose LINC01833 expression was less than the average were placed in the other group, referred to as the low expression group. As exhibited in Table 1, data based on chi-square tests revealed that high expression of LINC01833 was associated with gender and N stage. However, there was no significant difference in age, T stage, or pathologic stage (all *p* > 0.05).

### 3.3. The Prognostic Value of LINC01833 Expression in LUAD Patients

**Figure 3:**
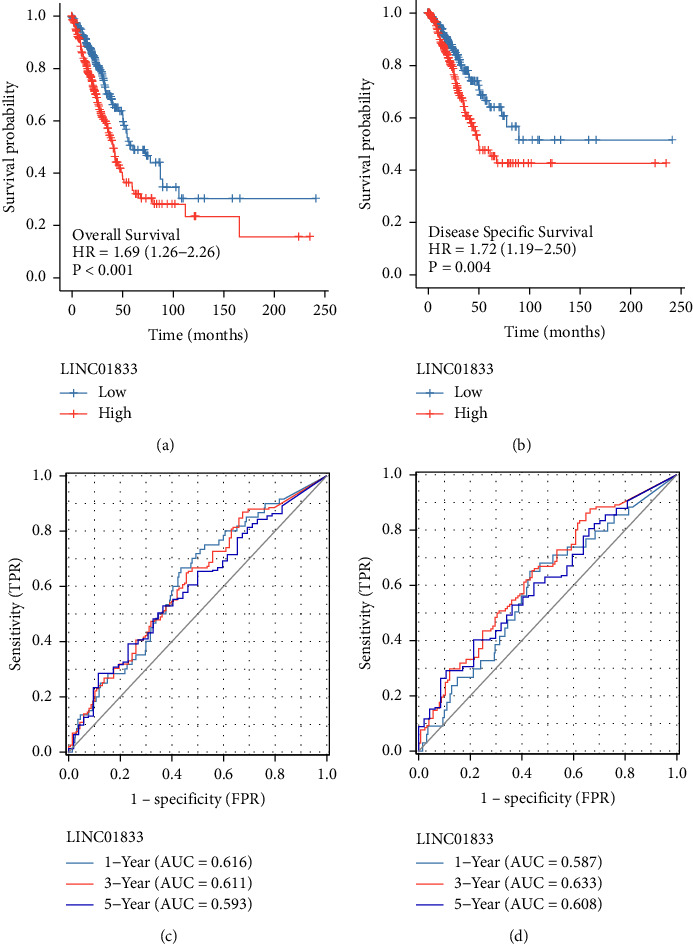
Survival assays of LINC01833 expression in LUAD patients. ((a) and (b)) Kaplan–Meier curves of OS and DSS. ((c) and (d)) Time-dependent ROC for LINC01833 expression in OS and DSS.

We employed Kaplan–Meier survival and log-rank analysis to further study the link between LINC01833 expression and the survival of LUAD patients. Specifically, we were interested in determining whether or not there was a significant difference. As shown in Figures 3(a) and 3(b), we found that patients with high LINC01833 expression exhibited shorter OS and DSS than those with low LINC01833 expression. In addition, the AUCs of the OS projected value in one year, two years, and three years were 0.616, 0.611, and 0.593, respectively, as determined by a time-dependent ROC analysis (Figure 3(c)). And the AUCs for the DSS prediction over one, two, and three years were 0.587, 0.633, and 0.608, respectively (Figure 3(d)). The results showed that the discrimination of LINC01833 expression was good. More importantly, we performed Cox proportional hazards regression analysis to explore the effects of LINC01833, as well as clinicopathological factors, on patient survival. As shown in Table 2, we found that pathologic stage and LINC01833 expression were independent prognostic factors for OS in LUAD patients (all *p* < 0.001). Moreover, a similar finding was also observed in the DSS of LUAD patients (Table 3).

### 3.4. Immune Cell Infiltration in LUAD Tissues

**Figure 4:**
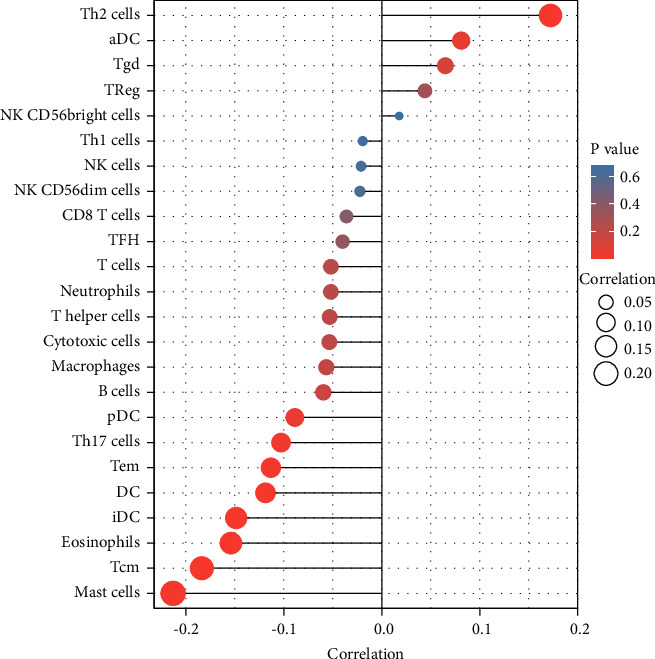
The correlation between LINC01833 expression and immune infiltration level in LUAD.

Immune infiltration into the microenvironment of the tumor is an important component in determining the efficacy of anticancer treatment and the outcome for the patient [26]. We investigated whether or not there was a link between the expression of LINC01833 and immune infiltration characteristics in LUAD tissues. As shown in Figure 4, we found that the expression of LINC01833 was positively associated with Th2 cells, aDC, and Tgd, while negatively associated with Mast cells, Tcm, Eosinophils, iDC, DC, Tem, Th17 cells, and pDC.

## 4. Discussion

Accumulating evidence has accumulated to suggest that aberrant expressions of lncRNAs can function either as oncogenes or tumor suppressors in a variety of malignancies [16, 27]. By interacting with DNA, RNA, or proteins, it has been established that lncRNA can perform the functions of molecular scaffolds, sponges, or coactivators [28, 29]. There are numerous lncRNAs that are involved in the development of tumors, playing important parts in the processes of tumor proliferation, invasion, and metastasis [30, 31]. Therefore, the identification of tumor-related lncRNAs is vital for both giving prospective therapeutic options for patients with cancer and understanding their function in the process of carcinogenesis.

In recent years, a number of researchers have confirmed that the clinicopathological features of a patient's smoking history, sex, age, pathological stage, distant organ metastasis, lymph node metastasis, and tumor size exhibit a distinct value in predicting the clinical outcome of LUAD patients [32–34]. A positive connection between lncRNAs and clinicopathological characteristics has been revealed by an increasing number of studies. In addition, in vitro and in vivo experiments also confirmed that lncRNAs served as tumor promoters or suppressors in tumor progression. For instance, Luo et al. reported that MCM3AP-AS1 was increased in individuals diagnosed with SCLC, and a high MCM3AP-AS1 level was associated with a decreased likelihood of survival. Overexpression of MCM3AP-AS1 increased cancer cell invasion and migration via sponging of the miR-148a pathway [35]. Chang et al. showed that lncRNA ITGB1-DT was shown to be elevated in LUAD, and high expression of ITGB1-DT was found to be connected with advanced clinical stages as well as poor overall survival and disease-free survival. Through the formation of a positive feedback loop with ITGB1/Wnt/-catenin/MYC, increased expression of ITGB1-DT made it easier for LUAD cells to proliferate, migrate, and invade [36]. This also made lung metastasis in vivo more likely to occur. These findings suggested that lncRNAs may be used as novel biomarkers for LUAD patients. In this study, we found a new lncRNA called LINC01833 that was associated with LUAD and found that its expression was much higher in LUAD samples than in nontumor samples. Additionally, ROC assays indicated that LINC01833 had a good capacity for screening LUAD specimens from nontumor specimens, which suggested that LINC01833 might be used as a potential diagnostic biomarker for LUAD. After that, we discovered that a high level of LINC01833 expression was connected to a bad prognosis for LUAD patients. It is important to note that multivariate analyses indicated that LINC01833 expression acted as an independent predictive factor for overall survival and disease-specific survival in LUAD patients. Based on our findings, LINC01833 may serve as a unique diagnostic and prognostic biomarker for patients suffering from LUAD.

The tumor immune microenvironment (TIME) is now thought to have a substantial impact on the clinical treatment response and prognosis of patients who have tumors, thanks to the development of technologies that are both high-throughput and accurate in their detecting capabilities [37, 38]. It has been proven that a positive correlation exists between immune cell infiltration in tumors and the prognosis of LUAD. Based on the above results, we hypothesize that the TIME status is a promising indication of the response to LUAD treatment and the prognosis of the patient [39, 40]. In this study, we found that the expression of LINC01833 was positively associated with Th2 cells, aDC, and Tgd, while negatively associated with Mast cells, Tcm, Eosinophils, iDC, DC, Tem, Th17 cells, and pDC. It has been demonstrated that Th2 and Th17 cells, as well as some other immune cells, play an important role in LUAD progression [41, 42]. Our findings suggested that LINC01833 may be a potential prognostic marker and a therapeutic target of the TME in LUAD.

However, several limitations in this study should be noted. Firstly, it is vital to incorporate several clinical parameters of patients who are receiving LUAD treatment in order to properly illuminate the precise role that LINC01833 played in the development of LUAD. It is necessary to include multiple clinical parameters for patients receiving LUAD treatment. Second, further molecular tests were warranted in order to validate the mechanisms of LINC01833 and its impact on the clinical outcome in LUAD. In addition, it is essential to integrate and elaborate on the link between LINC01833 and chemokines/chemokine receptors because this can assist us in gaining a deeper comprehension of the TME, particularly the immune microenvironment in tumors.

## 5. Conclusion

In summary, elevated LINC01833 expression was correlated with poor prognosis and positively associated with Th2 cells, aDC, and Tgd, while negatively associated with Mast cells, Tcm, Eosinophils, iDC, DC, Tem, Th17 cells, and pDC. Therefore, LINC01833 may serve as a prognostic biomarker for LUAD in addition to playing a significant role in immune cell infiltration.

## Figures and Tables

**Table 1:** Clinicopathological features and LINC01833 expression in LUAD.

Characteristics	Low expression of LINC01833	High expression of LINC01833	*p*
*n*	267	268	
Gender, *n* (%)			0.034
Female	130 (24.3%)	156 (29.2%)	
Male	137 (25.6%)	112 (20.9%)	
Age, *n* (%)			0.860
≤65	126 (24.4%)	129 (25%)	
>65	132 (25.6%)	129 (25%)	
T stage, *n* (%)			0.316
T1	97 (18.2%)	78 (14.7%)	
T2	139 (26.1%)	150 (28.2%)	
T3	21 (3.9%)	28 (5.3%)	
T4	9 (1.7%)	10 (1.9%)	
N stage, *n* (%)			0.045
N0	185 (35.6%)	163 (31.4%)	
N1	43 (8.3%)	52 (10%)	
N2	29 (5.6%)	45 (8.7%)	
N3	0 (0%)	2 (0.4%)	
M stage, *n* (%)			1.000
M0	180 (46.6%)	181 (46.9%)	
M1	12 (3.1%)	13 (3.4%)	
Pathologic stage, *n* (%)			0.069
Stage I	159 (30.2%)	135 (25.6%)	
Stage II	55 (10.4%)	68 (12.9%)	
Stage III	33 (6.3%)	51 (9.7%)	
Stage IV	13 (2.5%)	13 (2.5%)	
Age, median (IQR)	66 (59, 72)	65.5 (59, 73)	0.791

**Table 2:** Univariate and multivariate analyses for overall survival.

Characteristics	Total (*N*)	Univariate analysis		Multivariate analysis	
		Hazard ratio (95% CI)	*p* value	Hazard ratio (95% CI)	*p* value
Gender	526				
Female	280	Reference			
Male	246	1.070 (0.803–1.426)	0.642		
Age	516				
≤65	255	Reference			
>65	261	1.223 (0.916–1.635)	0.172		
Pathologic stage	518				
Stage I and stage II	411	Reference			
Stage III and stage IV	107	2.664 (1.960–3.621)	**<0.001**	2.574 (1.892–3.503)	**<0.001**
LINC01833	526				
Low	261	Reference			
High	265	1.686 (1.259–2.257)	**<0.001**	1.641 (1.222–2.205)	**0.001**

**Table 3:** Univariate and multivariate analyses for disease-specific survival.

Characteristics	Total (*N*)	Univariate analysis		Multivariate analysis	
		Hazard ratio (95% CI)	*p* value	Hazard ratio (95% CI)	*p* value
Gender	491				
Female	262	Reference			
Male	229	0.989 (0.687–1.424)	0.954		
Age	481				
≤65	243	Reference			
>65	238	1.013 (0.701–1.464)	0.944		
Pathologic stage	483				
Stage I and stage II	389	Reference			
Stage III and stage IV	94	2.436 (1.645–3.605)	**<0.001**	2.323 (1.567–3.443)	**<0.001**
LINC01833	491				
Low	246	Reference			
High	245	1.725 (1.191–2.497)	**0.004**	1.689 (1.159–2.462)	**0.006**

## Data Availability

The datasets used and analyzed during the current study are available from the corresponding authors upon reasonable request.
